# Validation of the EQ-5D-Y-5L parent-proxy version among children with juvenile idiopathic arthritis

**DOI:** 10.1007/s11136-024-03682-4

**Published:** 2024-08-14

**Authors:** Arto Ohinmaa, Jiabi Wen, Gillian R. Currie, Susanne M Benseler, Joost F Swart, Sebastiaan J Vastert, Rae S M Yeung, Deborah A. Marshall

**Affiliations:** 1 School of Public Health, University of Alberta, Edmonton, Canada; 2 Department of Paediatrics, Cumming School of Medicine, University of Calgary, Calgary, Canada; 3 Department of Community Health Sciences, Cumming School of Medicine, University of Calgary, Calgary, Canada; 4 Alberta Children’s Hospital Research Institute, University of Calgary, Calgary, Canada; 5 Department of Pediatric Immunology and Rheumatology, Wilhelmina Children’s Hospital / UMC Utrecht and University of Utrecht, Utrecht, The Netherlands; 6 Department of Paediatrics, Immunology and Medical Science, The Hospital for Sick Children, University of Toronto, Toronto, Canada; 7 McCaig Institute for Bone and Joint Health, University of Calgary, Calgary, Canada; 8 O’Brien Institute for Public Health, University of Calgary, Calgary, Canada

**Keywords:** EQ-5D-Y-5L, Validity, Juvenile idiopathic arthritis, Psychometric properties, Patient-reported outcome measure, Health-related quality of life

## Abstract

**Objectives:**

Juvenile idiopathic arthritis (JIA) is the most common type of arthritis among children. It can cause joint pain and permanent physical damage, which affects mobility and daily activities. The EQ-5D-Y-3L self-report version has been validated in JIA, but the validity of EQ-5D-Y-5L remains unknown. We examined the psychometric properties of the EQ-5D-Y-5L parent-proxy version among children with JIA.

**Methods:**

We used data from the Understanding Childhood Arthritis Network Canadian-Dutch collaboration study cohort, including patients with new-onset JIA, and those starting or stopping biologics. Clinical data and the parent-proxy version of the childhood health assessment questionnaire (CHAQ) and EQ-5D-Y-5L were collected. We evaluated the ceiling and floor effect; convergent and divergent validity using Spearman’s rank correlation; known-group validity using one-way ANOVA (Analysis of Variance) and effect size; and informativity using Shannon’s evenness index.

**Results:**

467 patient visits representing 407 patients were analyzed. The EQ-5D-Y-5L had no ceiling/floor effect. The EQ-5D-Y-5L showed good convergent (e.g., EQ-5D-Y-5L pain/discomfort dimension vs. CHAQ pain index (Spearman’s *r* = 0.74, 95% confidence interval (C.I.): 0.69–0.79)), divergent (e.g., EQ-5D-Y-5L pain/discomfort dimension vs. CHAQ eating dimension (Spearman’s *r* = 0.19, 95% C.I.: 0.09–0.29)) and known-group validity (e.g., mean EQ-5D-Y-5L level summary score for patients with inactive versus active disease status, 6.34 vs. 10.52 (*p* < 0.001, effect size = 1.20 (95% C.I.: 0.95–1.45)). Shannon’s evenness index ranged from 0.52 to 0.88, suggesting most dimensions had relatively even distributions.

**Conclusions:**

In this patient sample, EQ-5D-Y-5L parent-proxy version exhibited construct validity and informativity, suggesting the EQ-5D-Y-5L can be used to measure the quality of life of children with JIA.

**Supplementary Information:**

The online version contains supplementary material available at 10.1007/s11136-024-03682-4.

## Abstract (plain language summary)

Juvenile idiopathic arthritis is the most common type of arthritis affecting children. It can cause pain and permanent physical damage to joints and affects mobility and daily activities. While there is no cure yet, new therapies like biologics are effective. However, biologics are expensive and can have side effects. To decide when is the best time to use these biologics, we need to understand their cost and impact on patients. EQ-5D-Y-5L is a common tool to measure how the disease affects a patient’s life. It is unclear whether EQ-5D-Y-5L works well for patients with juvenile idiopathic arthritis. In this study, we compared the EQ-5D-Y-5L to another tool that measures how the illness impacts functional ability. We looked to see if the EQ-5D-Y-5L could tell the difference between children who were more or less sick. We also assessed whether the EQ-5D-Y-5L has the ability to describe patients with different severity in health status. This study indicates that the EQ-5D-Y-5L is a good tool to measure the health of patients with juvenile idiopathic arthritis. Findings from this study support the use of the EQ-5D-Y-5L among this patient population in future clinical trials and research studies.

**Supplementary Information:**

The online version contains supplementary material available at 10.1007/s11136-024-03682-4.

## Introduction

Juvenile idiopathic arthritis (JIA) encompasses all forms of arthritis with unknown causes starting before 16 years old [1, 2]. It is the most common chronic rheumatic disease in children [1, 2], affecting approximately 1 per 1,000 Canadian children and 0.07–4.01 per 1,000 children worldwide [3, 4]. Different subtypes vary in clinical presentation, pathogenesis, and prognosis; however, they have common symptoms, such as morning joint stiffness, joint pain, and joint swelling [5]. If untreated, JIA can lead to joint damage, functional limitation, and severe disability [6]. There is no cure, and treatment mainly manages pain and inflammation through a combination of pharmacological, physical, and psychosocial therapies to control symptoms and avoid joint damage [4, 7]. Among pharmaceutical treatments, the relatively-new biologic disease-modifying antirheumatic drugs, commonly referred to as biologics, have shown high efficacy for disease remission and preventing long-term disability, however, they have side effects, high costs, and unknown long-term safety [7, 8].

Patient-reported outcome measures (PROMs) are used to assess functional status, pain, and health-related quality of life (HRQL) for JIA patients in clinical settings. They provide an inclusive picture of health status and help clinicians understand the impact of JIA on children’s everyday life and the life course [9–11]. Measuring PROMs reflects whether clinical care makes patients feel better [12, 13]. The commonly used JIA-specific PROMs, such as the Childhood Health Assessment Questionnaire (CHAQ), incorporate outcomes of patients’ direct interest, and can provide an effective measurement of health status and predict patient outcomes [12, 14, 15].

Several generic pediatric HRQL measures including the EQ-5D-Y-3L have been validated and used among children with JIA [16]. Valuation studies on the EQ-5D-Y-3L, published or underway, support the use of EQ-5D-Y-3L in future economic evaluation in JIA, which is important given the high costs of JIA treatments [16, 17]. The EQ-5D-Y-3L has only 3 levels in each dimension, potentially leading to large ceiling effects [18]. The EQ-5D-Y-5L, still experimental, is a 5-level version revised from the EQ-5D-Y-3L that aims to reduce ceiling effects and enhance sensitivity [19]. It has been validated in several disease areas and the general population, mainly among children aged 8 years old or more, via the self-report or the caregiver-proxy (reported by a caregiver on behalf of the children) version, and found to have less ceiling effects, better responsiveness and discriminate power (versus the EQ-5D-Y-3L), good test-retest reliability, convergent validity, and known-group validity [20–27]. Among studies exploring the psychometric properties of the caregiver-proxy version, good agreement between the proxy and the self-report version was identified [25, 26]. There remains a knowledge gap in the performance of EQ-5D-Y-5L among children under 8 years old, and the psychometric properties of the EQ-5D-Y-5L among patients with JIA of all ages.

As young children have difficulty completing the PROM by themselves, a proxy version is required. For both the CHAQ and EQ-5D-Y, the age threshold for mandatory proxy-version is 8 years [28, 29]. Many JIA patients have the disease onset at a very young age (2–4 years) [30]. Valid tools are necessary to assess their HRQL before age 8.

This paper focuses on assessing the psychometric properties of the parent-proxy version of the EQ-5D-Y-5L among patients with JIA in terms of its ceiling effect, construct validity (convergent validity, divergent validity, and known-group validity), and informativity.

## Methods

### Data

This validation study used data collected from the Understanding Childhood Arthritis Network (UCAN) Canadian-Dutch collaboration (CAN-DU) [31] study cohort between October 2019 and May 2023. Three cohorts were analyzed in this study, patients with new-onset JIA, patients starting or restarting biologics, and patients stopping biologics. Depending on disease progression, each study participant could be in several cohorts throughout the study period. In each cohort, patients have a baseline clinical visit and at least one follow-up visit. Clinical data, and the parent-proxy version of the CHAQ and the EQ-5D-Y-5L were collected during each visit. The analytical sample for this cross-sectional validation comprised data from all baseline visits where EQ-5D-Y-5L data was collected within a 30-day window. For clinical data and CHAQ with missing values, we perform analyses pertaining a specific variable with the complete dataset for that variable and report the extent of missing values. Ethics approval was granted by the Conjoint Health Research Ethics Board at the University of Calgary (REB17–1563), the Health Research Ethics Board at the University of Alberta (Pro00106423), and the Ethical Board of Utrecht (18–474).

### Measures

#### Clinical measures

Patient age, biological sex, country of residence (Canada, Netherlands), cohort^1^, JIA subtypes^2^, disease duration at the time of visit (up to 12 months before this visit, more than 12 months before this visit) were collected. We used several disease severity and disease activity measures including disease status assessed by physicians (active, inactive), active joint count, presence of morning joint stiffness, presence of joint pain, physician global assessment of disease activity (10-point VAS), and disease activity (inactive disease, minimal disease activity, moderate disease activity, and high disease activity) assessed by the clinical juvenile arthritis disease activity score-10 (cJADAS10, a composite disease activity score for JIA calculated from physician’s global rating of overall disease activity, parent/child ratings of well-being, and count of active joints) [32, 33].

#### EQ-5D-Y-5L parent-proxy version

The EQ-5D-Y-5L [19] is a generic HRQL measure for the young population. The descriptive system measures the health status of “today” through five dimensions including mobility, looking after myself, doing usual activities, having pain or discomfort, and feeling worried, sad, or unhappy and a vertical visual analog scale (VAS) ranging from 0 (the worst health) to 100 (the best health). The EQ-5D-Y-5L has five levels: 1 “no problems”, 2 “a little bit of problems”, 3 “some problems”, 4 “a lot of problems” and 5 “extreme problems/cannot do” [34]. There is currently no value set for the EQ-5D-Y-5L to summarize the five dimensions to a single preference-based index. An alternative method to summarize the descriptive system is by calculating a level summary score (ranging from 5 to 25), which is a total sum score of the severity levels of each dimension [35, 36].

There is no current official recommendation regarding the user age range in the administration of the EQ-5D-Y-5L. For EQ-5D-Y-3L, it is not applicable to children aged 0–3 years old, and a proxy version should be used for children aged 4–7 years old. As our study population includes children aged 4–7 years, we used the parent-proxy version of EQ-5D-Y-5L and CHAQ for all ages for this paper.

#### CHAQ parent-proxy version

The CHAQ [37] is a validated measure for children with JIA. It is designed to measure health status “over the past week”. The CHAQ includes a disability section, a discomfort section, and an overall assessment of the health status affected by arthritis. The disability section has eight dimensions: dressing and grooming, arising, eating, walking, hygiene, reach, grip, and activities. The dimensional score is determined by selecting the highest score among all items within each dimension. The item score ranges from 0 to 3: 0 “no difficulty”, 1 “some difficulty”, 2 “much difficulty”, 3 “unable to do”. If the use of aids or devices is reported for items within a particular dimension, the minimum score would be 2. The average score of all eight dimensions is the disability index (0–3, higher score reflecting greater disability). In the discomfort section, a horizontal VAS (0-100) is used to measure the presence of pain and its severity, with 0 representing “no pain” and 100 representing “very severe pain”. In the overall health status section, a horizontal VAS is used, with 0 representing “very well” and 100 representing “very poor”.

### Measurement properties

We performed descriptive analyses to describe the distribution of the EQ-5D-Y-5L. We estimated the mean (standard deviation, SD) and median (interquartile range, IQR) of the level summary score and explored the distribution of responses to each dimension. We analyzed the ceiling effect (assessed by the proportion of respondents reporting “no problems” in all dimensions) and floor effect (the proportion of respondents reporting “extreme problems/cannot do” in all dimensions). No ceiling/floor effect of the EQ-5D-Y-5L reflects less responses clustered on the upper (the best health) or lower (the worst health) bounds of the measurement scale, indicating ability to measure a wide range of values. A threshold of 15% was used to determine whether ceiling/floor effect was present [38].

Construct validity reflects the degree to which a measure really evaluates the construct to be measured [39]. We assessed the construct validity of the EQ-5D-Y-5L by exploring its convergent validity, divergent validity, and known-group validity. The convergent validity reflects that constructs theoretically the same or similar are in fact highly correlated with each other, while the divergent validity demonstrates that constructs theoretically not similar are in fact not highly correlated with each other [40, 41]. Demonstrative figures on the theoretical relationship between the dimensions of EQ-5D-Y-5L and the CHAQ were provided in Appendix 1. We used Spearman’s rank correlation (Spearman’s r) to explore the association between the EQ-5D-Y-5L and CHAQ dimensions. The strength of the correlation was interpreted using the following criteria: no (*r* < 0.2), weak (0.2 ≤ *r* < 0.35), moderate (0.35 ≤ *r* < 0.5), and strong (*r* ≥ 0.5) [42].

Known-group validity reflects whether a measure can distinguish between two or more groups that are known to differ in the variable of interest [43]. Known-group validity can be considered present when at least 75% of the results are in correspondence with the hypotheses, in groups of at least 50 patients [38]. Studies have found that suboptimal HRQL among patients with JIA was associated with non-oligoarthritis [37, 44–46], shorter disease duration [45, 47], severe disease activity [45, 48–50], higher level of pain [45, 48, 49], severe functional disability [45, 48, 49], and poor well-being [48, 49]. Based on these studies, the formation of known groups with our study data was presented in Appendix 2. We used the one-way analysis of variance (ANOVA) to test the mean difference in EQ-5D-Y-5L level summary score across each known group. When the known group has three or more levels, each level was compared with the one subsequently less severe^3^, and a Bonferroni correction was used in these post-hoc pairwise comparisons. In total, 17 hypotheses across 11 known-group variables were examined with the statistical significance level being *p* < 0.05; the above noted 75% threshold can be applied to this to draw conclusions about known group validity. We also estimated the effect sizes (Cohen’s d) to quantify the magnitude of mean difference across each known group. The effect size was estimated by the mean difference divided by the pooled standard deviation. The magnitude of the effect size was interpreted as: 0.2 to 0.49, small; 0.5 to 0.79, moderate; and ≥ 0.8, large [51].

Post-hoc power analysis indicated sufficient power to detect all effect sizes of interest (i.e., *r* = 0.35 for correlations between EQ-5D-Y-5L and CHAQ for construct validity and effect size (*d)* = 0.5 for known groups differences).

Informativity reflects the ability of a measure or item to discriminate between people with different characteristics measured by that item [52]. According to information theory, the informativity of an item is better when it has more categories and responses to this item are more evenly distributed among categories [53]. Shannon’s absolute index (H’) can express the extent to which the information is evenly distributed across categories, and Shannon’s evenness index (J’, defined by H’/H’_max_, i.e., the use of the system (H’) given its potential (H’_max_)) can express the relative evenness of the distribution, regardless of categories [53, 54]. Shannon’s J’ ranges from 0 to 1, with 1 indicating the item is perfectly even.

### Sensitivity analysis

Although the EQ-5D-Y-5L parent-proxy version is not recommended for children aged under 4 years old, this study recruited and administered the instrument with participants younger than 4 years. In the primary analysis, we analyzed the psychometric properties among children greater than 4 years old, and in the sensitivity analysis, we re-ran the analysis including children younger than 4.

## Results

### Patient characteristics

In the base case, 407 patients were included, representing 467 patient visits. At the entry to the UCAN CAN-DU study, patients had a median age of 12 years (interquartile range (IQR): 9–15), with 58% being female and 34% residing in Canada. Of all patient visits, 17% came from patients aged 4–7 years old; 45% were in the “new onset of JIA” cohort and 40% in the “starting biologics” cohort; 40% were characterized by the three oligoarticular subtypes and 25% by the two polyarticular subtypes; 41% had disease duration more than one year; 83% were characterized by an active disease status; 30% had the presence of morning joint stiffness (≥ 15 min); 31% had the presence of joint pain; and 59% were classified as moderate (39%) to severe (20%) disease activity using cJADAS10 scores. The median number of active joint counts was 2 (IQR: 1–5), and the median physician global assessment disease activity score was 2.5 (IQR: 1.3-4.0), with 0 indicating no activity. Among these variables, JIA subtypes (9%), disease duration (10%), active joint counts (2%), and cJADAS10 (23%) had missing values, with cJADAS10 having the highest missing rate (Table 1). Given that cJADAS10 measures disease activity, we used the binary disease activity variable to examine the characteristics of missing data, and we found that patients with inactive disease were more likely to have missing values in cJADAS10 (data not shown).

**Table 1:** Patient characteristics

	Base-case (excluding aged 1–3) Patient visits = 467; Patient *N* = 407	Sensitivity (including aged 1–3) Patient visits = 540; Patient *N* = 472
Age when entering the study, median (IQR), years	12 (9–15)	11 (6–14)
Age 4–7 years old, n (%)	81 (17%)	81 (15%)
Female, n (%)	236 (58%)	289 (61%)
Country of residence, n (%)		
Canada	137 (34%)	167 (35%)
Netherlands	270 (66%)	305 (65%)
Cohort, n (%) ^1^		
New onset of JIA	222 (45%)	277 (49%)
Start biologics	196 (40%)	216 (38%)
Stop biologics	71 (15%)	73 (13%)
JIA classification ^2^, n (%)		
Extended Oligoarticular JIA	36 (8%)	38 (7%)
Persistent Oligoarticular JIA	65 (14%)	71 (13%)
Oligoarticular JIA (not classified yet: < 6 months)	86 (18%)	122 (23%)
Polyarticular JIA RF negative	93 (20%)	104 (19%)
Polyarticular JIA RF positive	23 (5%)	23 (4%)
Enthesitis-related arthritis	61 (13%)	61 (11%)
Psoriatic arthritis	20 (4%)	23 (4%)
Undifferentiated JIA	18 (3%)	20 (4%)
Systemic JIA	23 (5%)	28 (5%)
Missing	42 (9%)	50 (9%)
Duration of disease at the time of visit, n (%)		
More than 12 months	193 (41%)	202 (37%)
Up to 12 months	227 (48%)	282 (52%)
Missing	47 (10%)	56 (10%)
Disease status, n (%)		
Inactive	79 (17%)	82 (15%)
Active	388 (83%)	458 (85%)
Active joint count, median (IQR)	2 (1–5)	2 (1–5)
No active joints, n (%)	96 (21%)	100 (19%)
1–5 active joints, n (%)	253 (54%)	310 (57%)
6 or more active joints, n (%)	111 (24%)	122 (23%)
Missing, n (%)	7 (2%)	8 (1%)
Morning joint stiffness ≥ 15 min, n (%)	139 (30%)	161 (30%)
Having joint pain, n (%)	146 (31%)	164 (30%)
Disease activity based on cJADAS10, n (%)		
Inactive disease	51 (11%)	53 (10%)
Minimal disease activity	32 (7%)	42 (8%)
Moderate disease activity	182 (39%)	216 (40%)
High disease activity	95 (20%)	109 (20%)
Missing	107 (23%)	120 (22%)
Physician Global Assessment of disease activity (PGA, 10-point VAS), median (IQR)	2.5 (1.3-4.0)	2.6 (1.5-4.0)
PGA: 0 (no activity)–- 0.9, n (%)	85 (18%)	88 (16%)
PGA: 1–4, n (%)	289 (62%)	340 (63%)
PGA: 4.1–10 (maximum activity), n (%)	93 (20%)	112 (21%)

Note: JIA Juvenile idiopathic arthritis, IQR Interquartile range, RF Rheumatoid factor

1. In each visit, a patient might be enrolled in multiple cohorts

2. Oligoarticular: number of affected joints < 5; Polyarticular: number of affected joints > 4; Systemic JIA: arthritis with systemic features; Enthesitis-related JIA: arthritis with the enthesitis features; Psoriatic JIA: arthritis with the psoriatic feature; undifferentiated JIA: does not fulfill the criteria for any JIA subtype or fulfills criteria for more than one subtype)

### Response pattern

**Fig. 1:**
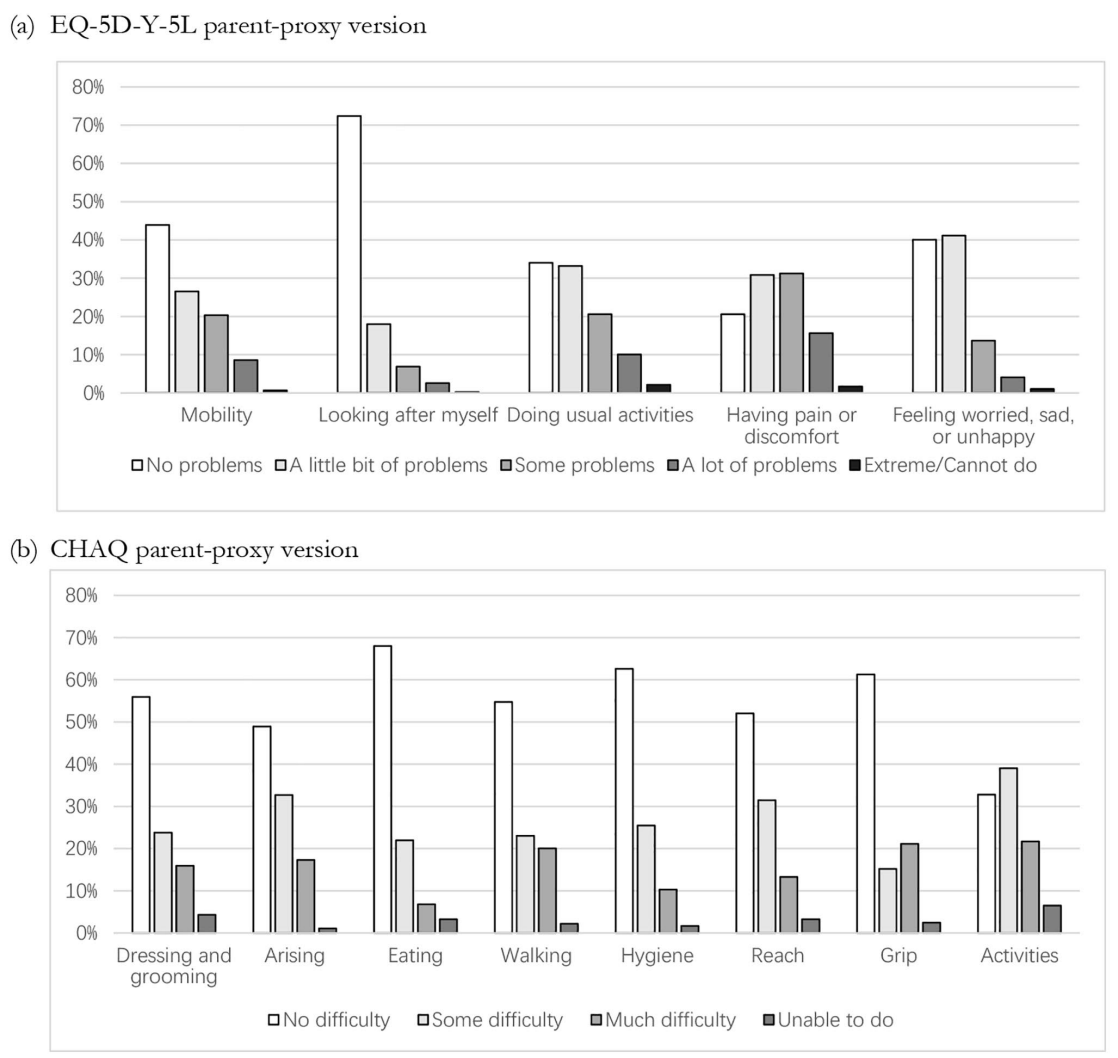
EQ-5D-Y-5L parent-proxy version and Childhood Health Assessment Questionnaire (CHAQ) parent-proxy version dimensional responses (Base case: 467 patient visits representing 407 patients)

The mean EQ-5D-Y-5L level summary score was 9.81 (SD = 3.81) and the median score was 9 (IQR: 7–12). The EQ-VAS score had an estimated mean of 70.67 (SD = 20.78) and a median of 74 (58–88). The percentage of responses reporting the best or worst possible EQ-5D-Y-5L profiles was 13% and 0%, respectively, suggesting no ceiling or floor effect effects (Table 2). The “having pain or discomfort” dimension had the most problems reported (79%), followed by “doing usual activities” (66%), the “feeling worried, sad, or unhappy” (60%), and the “mobility” dimension (56%). There were fewer problems in the “looking after myself” dimension (28%) compared to other dimensions (Fig. 1).

**Table 2:** Descriptive statistics of EQ-5D-Y-5L and CHAQ

		Base-case (excluding aged 1–3) Patient visits = 467; Patient *N* = 407	Sensitivity (including aged 1–3) Patient visits = 540; Patient *N* = 472
EQ-5D-Y-5L level summary score (LSS)	Mean (SD)	9.81 (3.81)	10.05 (3.98)
	Median (IQR)	9 (7–12)	10 (7–12)
	Missing, n	0	0
Skewness, Kurtosis		0.76, 3.14	0.78, 3.16
Number of “11111” ^1^	n (%)	60 (13%)	64 (12%)
Number of “55555” ^2^	n (%)	0 (0%)	0 (0%)
EQ-VAS	Mean (SD)	70.67 (20.78)	70.92 (20.45)
	Median (IQR)	74 (58–88)	75 (59–87)
	Missing, n	0	0
CHAQ disability index	Mean (SD)	0.68 (0.62)	0.68 (0.62)
	Median (IQR)	0.5 (0.1-1)	0.5 (0.1-1)
	Missing, n (%)	97 (21%)	107 (20%)
	Association with LSS	0.77	0.72
CHAQ pain index	Mean (SD)	41.44 (28.95)	41.20 (28.82)
	Median (IQR)	41 (13–68)	40 (13–66)
	Missing, n (%)	97 (21%)	107 (20%)
CHAQ health status	Mean (SD)	37.77 (28.73)	37.17 (28.43)
	Median (IQR)	37 (10–62)	35 (10–61)
	Missing, n (%)	98 (21%)	108 (20%)
	Association with EQ-VAS	-0.65	-0.65

Note: VAS visual analog scale, SD standard deviation, IQR interquartile range

1. An “11111” EQ-5D-Y-5L profile: reporting “no problems” in all five EQ-5D-Y-5 L dimensions

2. A “55555” EQ-5D-Y-5L profile: reporting “extreme problems/cannot do” in all five EQ-5D-Y-5L dimensions

The CHAQ disability index had a mean and median of 0.68 (SD = 0.62) and 0.5 (IQR: 0.1-1), respectively, and was strongly correlated with the EQ-5D-Y-5L level summary score (*r* = 0.77). Of all dimensions, patients reported greater disability to perform tasks in the “activities” dimension, with only 33% reporting no difficulty. The “dressing and grooming”, “arising”, “walking”, and “reach” dimensions had higher proportions reporting no difficulty (49–56%), with the “eating”, “hygiene”, and “grip” dimensions having the highest proportions reporting no difficulty (61-68%). The CHAQ pain index had an estimated mean of 41.44 (SD = 28.95) and median of 41 (IQR: 13–68), and the CHAQ health status index had an estimated mean of 37.77 (SD = 28.73) and median of 37 (IQR: 10–62). For both indices, 0 represents the best possible situation. The CHAQ health status strongly correlated with the EQ-VAS (*r*=-0.65) (Table 2; Fig. 1). The CHAQ had a missing rate of 21%. Characteristics of missing data were examined using the EQ-5D-Y-5L level summary score and EQ-VAS. Patients with missing values in CHAQ tended to have EQ-5D-Y-5L lower level summary scores and higher EQ-VAS scores, indicating, better HRQL status (data not shown).

### Convergent and divergent validity

EQ-5D-Y-5L dimensions that theoretically measure the similar or same constructs as the CHAQ dimensions, demonstrated strong associations (*r* = 0.59–0.74). The exception was the EQ-5D-Y-5L “doing usual activities” versus the CHAQ “grip” dimension which was moderately correlated (*r* = 0.37, 95% confidence interval: 0.28–0.46). This suggests good convergent validity for the EQ-5D-Y-5L parent-proxy version. For EQ-5D-Y-5L dimensions that theoretically might be correlated with the CHAQ dimensions, we also observed moderate to strong associations (*r* = 0.41–0.65), except for EQ-5D-Y-5L “having pain or discomfort” versus the CHAQ “grip” dimension (*r* = 0.33, 95% confidence interval: 0.24–0.42) (Table 3).

**Table 3:** Convergent-divergent validity results: Correlation between EQ-5D-Y-5L and CHAQ dimensions, presented by “theoretically strongly correlated”, “theoretically moderately correlated”, and “theoretically weakly correlated or no correlation” categories (Base-case: 370 patient visits representing 329 patients)

	Dressing and grooming	Arising	Eating	Walking	Hygiene	Reach	Grip	Activities	Pain index
**Theoretically strongly correlated**									
Mobility		**0.61** (0.54–0.66)		**0.69** (0.64–0.74)				**0.65** (0.59–0.70)	
Looking after myself	**0.69** (0.64–0.74)				**0.63** (0.56–0.69)	**0.59** (0.52–0.66)			
Doing usual activities						**0.53** (0.46–0.60)	0.37 (0.28–0.46)	**0.69** (0.63–0.74)	
Having pain or discomfort									**0.74** (0.69–0.79)
Feeling worried, sad, or unhappy									
**Theoretically moderately correlated**									
Mobility					**0.50** (0.42–0.57)	0.44 (0.36–0.52)			**0.60** (0.53–0.66)
Looking after myself		**0.52** (0.44–0.59)	0.44 (0.35–0.52)	0.45 (0.37–0.53)			0.45 (0.36–0.53)		0.41 (0.33–0.49)
Doing usual activities	0.43 (0.34–0.51)	**0.59** (0.52–0.65)		**0.60** (0.53–0.66)	**0.51** (0.43–0.58)				**0.65** (0.59–0.70)
Having pain or discomfort		**0.56** (0.49–0.63)		**0.52** (0.44–0.59)		**0.50** (0.42–0.57)	*0.33* (0.24–0.42)	**0.59** (0.52–0.66)	
Feeling worried, sad, or unhappy									
**Theoretically weakly correlated or no correlation**									
Mobility	0.39 (0.30–0.47)		*0.10* (0.002-0.20)				*0.16* (0.06–0.26)		
Looking after myself								0.48 (0.40–0.56)	
Doing usual activities			*0.23* (0.13–0.33)						
Having pain or discomfort	0.39 (0.30–0.47)		*0.19* (0.09–0.29)		0.45 (0.36–0.53)				
Feeling worried, sad, or unhappy	*0.31* (0.22–0.40)	*0.32* (0.23–0.41)	*0.23* (0.14–0.33)	*0.31* (0.21–0.40)	*0.33* (0.23–0.41)	0.38 (0.30–0.47)	*0.34* (0.24–0.42)	0.41 (0.33–0.50)	0.45 (0.37–0.53)

Note: Bolded and underlined values represent strong statistical associations. Italicized values represent no to weak statistical associations. All Spearman’s correlation coefficients were significant at *p* < 0.001

For the EQ-5D-Y-5L dimensions that theoretically do not measure the similar constructs with the CHAQ dimensions, there was no association or weak to moderate associations (*r* = 0.10–0.48). No EQ-5D-Y-5L dimensions directly measure a similar construct as the CHAQ “eating” dimension, and only the “looking after myself” dimension might theoretically correlate with the “eating” dimension. The Spearman’s correlation between the “eating” dimension and each of the other four not-related EQ-5D-Y-5L dimensions reflected no association or weak association (*r* = 0.10–0.23). This indicated a good divergent validity (Table 3, Appendix 3).

### Known-group validity

Table 4 displays mean differences of EQ-5D-Y-5L level sum scores across Known Groups, their effect sizes, and *p*-values. Importantly, the distribution of the level sum scores was not normal. However, we decided to report means, SDs, and parametric metrics for three reasons. First, our reasonably large sample size makes the violation of the assumption of normality less problematic [55]. Second, the parametric metrics reported are common in the current literature and readers are more likely to be familiar with it, easing interpretation. Finally, we reached the same conclusions when comparing metrics from Table 3 with its non-parametric version that reports medians, IQR, median difference, Cliff’s delta, and non-parametric *p*-values (Appendix 4).

**Table 4:**
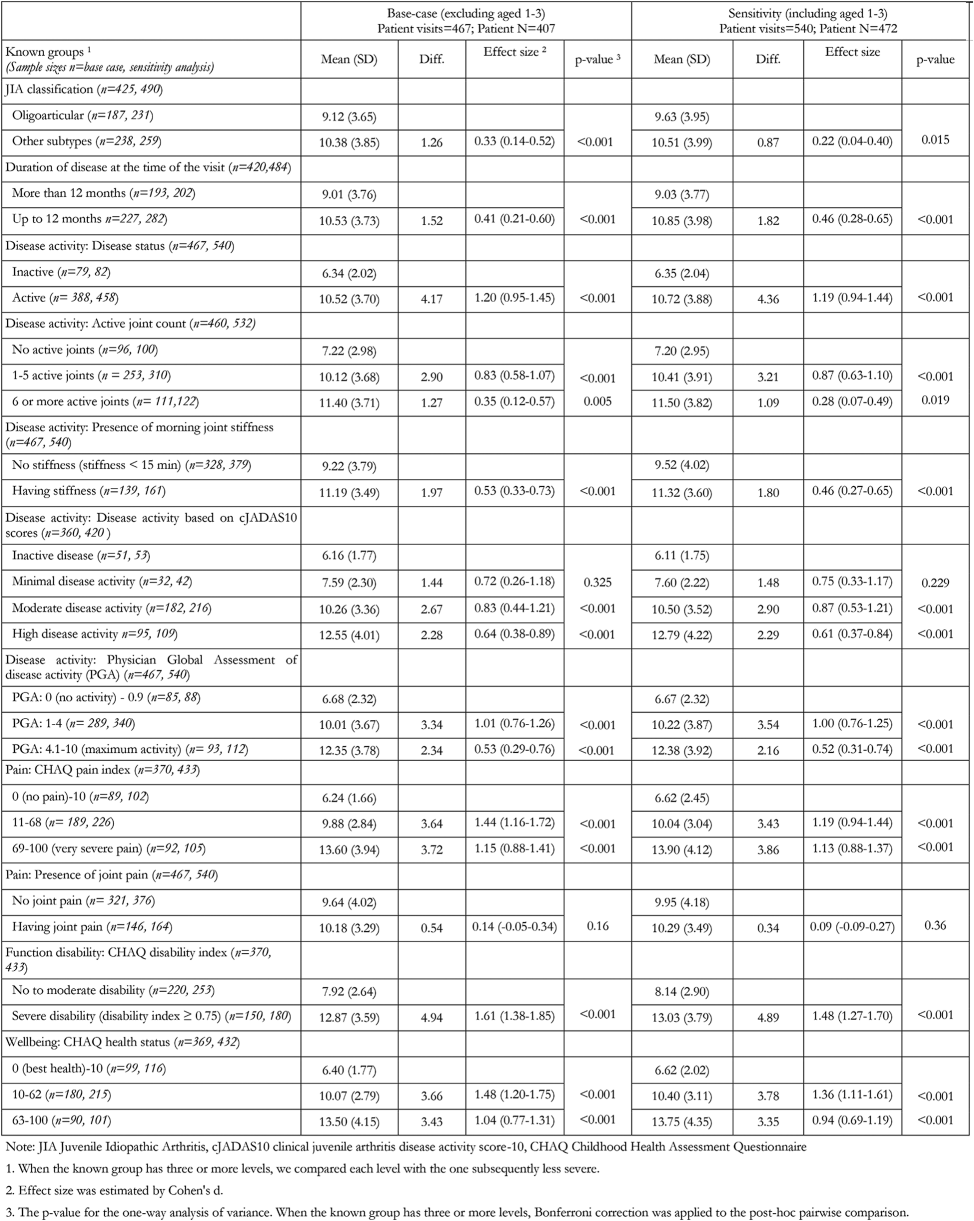
Known group analysis in terms of EQ-5D-Y-5L level summary scores

In the known-group analyses, patients with non-oligoarticular JIA, having shorter disease duration, experiencing more disease activity, suffering more pain, having more functional disabilities and worse well-being showed significantly higher EQ-5D-Y-5L level summary scores, indicating worse HRQL, compared to their counterparts. 15 of the 17 (88%) statistical tests were in accordance with the hypotheses at the *p* < 0.05 level, with 14 being significant at the *p* < 0.001 level, and one at *p* = 0.005 level (“1–5 active joints” vs. “6–10 active joints”). Comparisons between “minimal disease activity” and “no active disease” (*p* = 0.325) and between “having joint pain” and “no joint pain” (*p* = 0.16) were insignificant at the *p* < 0.05 level. The effect size of these mean differences was in moderate to large magnitude in most of the known groups, especially in the known groups related to disease activity, function disability, and wellbeing. This suggests that the EQ-5D-Y-5L parent-proxy version can discriminate pre-specified known groups (Table 4).

### Informativity

The Shannon’s evenness index for mobility, looking after myself, doing usual activities, having pain or discomfort, and feeling worried, sad, or unhappy dimensions were 0.80, 0.52, 0.85, 0.88, and 0.74, respectively. The informativity of looking after myself dimension was lower than the other dimensions (Table 5).

**Table 5:** Shannon’s absolute index (H’) and Shannon’s evenness index (J’)

	Base-case (excluding aged 1–3) Patient visits = 467; Patient *N* = 407			Sensitivity (including aged 1–3) Patient visits = 540; Patient *N* = 472	
	Shannon’s H’	Shannon’s J’		Shannon’s H’	Shannon’s J’
Mobility	1.28	0.80		1.32	0.82
Looking after myself	0.83	0.52		1.01	0.62
Doing usual activities	1.37	0.85		1.39	0.86
Having pain or discomfort	1.41	0.88		1.42	0.88
Feeling worried, sad, or unhappy	1.18	0.74		1.19	0.74

### Sensitivity analysis

In the sensitivity analyses, the sample had 472 patients representing 540 patient visits, with patients under 4 years old contributing 14% of patient visits. The patient characteristics of this sample were similar to the base case (Table 1). Slightly more problems were reported in the “looking after myself” dimension (33%) compared to the base case (28%), while the response pattern of the other dimensions and the summary score of the EQ-5D-Y-5L and EQ-VAS remained consistent (Table 2). The convergent, divergent, and known-group validity findings were also demonstrated in this sample (Appendix 3, Table 4). Compared with the base case, the Shannon’s evenness index showed similar values in all dimensions except the “looking after myself” dimension (0.62, versus base case 0.52) (Table 5).

## Discussion

This is the first study exploring the psychometric properties of the EQ-5D-Y-5L parent-proxy version among JIA patients. We used a heterogenous sample and found that the EQ-5D-Y-5L demonstrated a good convergent and divergent validity versus the CHAQ parent-proxy version and the ability to discriminate known groups defined by disease characteristics, functional ability, pain, and wellbeing, suggesting the future use of EQ-5D-Y-5L to assess HRQL for JIA patients.

We had a robust sample size representing a diverse range of patients. The base-case analytical sample included data from 467 visits representing 407 patients, which is more than previous EQ-5D-Y-5L validation studies [20–26]. The sample size for the correlation analysis was 370 visits, and ranged from360-467 for the known-group analyses. There was mild to moderate disease burden among the study population, based on various disease activity measures, and the functional ability, pain, and well-being assessed by the CHAQ. This is consistent with findings from other studies: the advancement of JIA management in recent years has led to a decrease in symptoms and disease activity and better overall assessment [16]. The HRQL measured by the EQ-5D-Y-5L reflects a similar pattern. The estimated mean and median of EQ-5D-Y-5L level summary score were around 9, which represents 4 dimensions with mild problems, 2 dimensions with moderate problems, or 1 dimension with severe problems (with the rest of the dimensions having no problems).

We examined the convergent and divergent validity of the EQ-5D-Y-5L by comparing its dimensions with the CHAQ dimensions. Each CHAQ dimension assesses the capability of a patient to perform tasks in that functional area. Only the “eating” dimension did not have a directly corresponding dimension in the EQ-5D-Y-5L that measured similar or the same constructs. For other functional areas, although some seem not semantically linked to any EQ-5D-Y-5L dimensions, e.g., “reach”, these dimensions had a wide range of tasks defining the functional ability, and it turns out that each dimension has one or more tasks reflecting similar constructs as the EQ-5D-Y-5L dimensions. In the “reach” dimension of the CHAQ, four tasks were included: (1) “Reach and get down a heavy object…”; (2) “Bend down to pick up…”; (3) Pull on a sweater over his/her head; (4) Turn neck to look back over shoulder. Based on the wording of these tasks, the “reach” dimension theoretically relates to the EQ-5D-Y-5L “looking after myself” and “doing usual activities” dimensions. Therefore, we were able to thoroughly analyze the convergent and divergent validity of the EQ-5D-Y-5L among JIA patients. The EQ-5D-Y-5L demonstrated good convergent and divergent validity. This reflected that though the EQ-5D-Y-5L, a generic measure, could not directly assess the JIA-specific functional ability, worse (or better) EQ-5D-Y-5L dimensional responses likely indicate that patients experience more (or less) difficulty in the corresponding functional areas.

We performed extensive known-group analyses to assess whether the EQ-5D-Y-5L could differentiate patients with known differences in HRQL, and the EQ-5D-Y-5L performed well in the specified known groups. 88% of the statistical tests were in accordance with the pre-specified hypotheses, surpassing the 75% threshold thus supporting that known-group validity is established [38]. In post-hoc pairwise comparisons where the known group contains three or more categories, Bonferroni correction was used, which reduced the chance of Type I error. Furthermore, the differences in HRQL across categories were significant at *p* < 0.001 level in most of the known groups, together with a moderate to large effect size.

Validation studies on the EQ-5D-Y-5L (not in the JIA population) made head-to-head comparison with the EQ-5D-Y-3L and show that the EQ-5D-Y-5L can decrease the ceiling effect and increase the informativity, compared with the EQ-5D-Y-3L [20–24, 26]. In our study, as the data collection is still ongoing and there is a limited sample with both instruments, we have not compared the 5L instrument with the 3L instrument, however, this can be explored in the next steps of this research. Using the 15% threshold [38], we found the EQ-5D-Y-5L has no ceiling/floor effect when measuring HRQL among patients with JIA. In terms of informativity, the reported Shannon’s evenness index in other EQ-5D-Y-5L validation studies ranged from 0.10 to 0.73 [20, 21, 24], and our study population reported 0.52–0.88, indicating more even distributions. Based on the ceiling/floor effect and the informativity statistics, the EQ-5D-Y-5L parent-proxy version has the informational richness to assess JIA patients.

JIA can be diagnosed at a very young age, and our study did recruit some patients younger than 4 years old. For these patients, EQ-5D-Y-5L is not recommended to use as some dimensions might not be appropriate for infants and toddlers. As such, we excluded children aged 1–3 years old in the base case and included them in the sensitivity analysis. We did not observe many differences in the psychometric properties between the two samples. Patients under 4 years old accounted for 14% of the sample, so this subpopulation possibly did not affect the overall results in a significant way. According to another study that solely analyzed the performance of the EQ-5D-Y-3L proxy version among young children (aged 3–5), HRQL expressed by the summary scores of the EQ-5D-Y-3L worked well and the known-group validity can be demonstrated in this young population. However, the “looking after myself” dimension is problematic for children aged 3 years old due to age-related difficulties [56]. This is consistent with our findings, as we also identified more problems reported in the “looking after myself” dimension in the sensitivity analysis. The EuroQol Research Foundation is developing EQ-TIPs [57], an instrument for infants aged 0–3 years old, to better measure HRQL among this population. The EQ-TIPS has an “eating” dimension, as eating is an important aspect of an infant and toddler’s life and a large focus of attention for parents [58]. Exploring the validity of EQ-TIPS on JIA patients is warranted considering the age characteristics of this population.

This study has strengths and limitations. The study sample was collected from national JIA cohorts in Canada and the Netherlands, which include many pediatric rheumatology clinics in these two countries. Recruited patients had various disease subtypes, were at different disease progression stages, and had diverse treatment experiences. There is also a great proportion of patients with relatively severe disease activities and disabilities. This suggests good generalizability of our study to JIA patients in western countries. However, we did not have enough data from patients aged 4–7 years old to analyze purely on this population. Patients aged 8 years and older dominated the study sample, and the psychometric performance of EQ-5D-Y-5L would be more determined by the older children. Therefore, the psychometric properties we observed might not provide the true picture among younger children. Also, the CHAQ does not have a dimension regarding mental health. We were unable to justify whether the “feeling worried, sad, or unhappy” dimension of the EQ-5D-Y-5L is valid to measure mental health conditions of the JIA population, although this dimension had a good divergent validity to all CHAQ dimensions.

Some clinical variables and the CHAQ used in the validation study had some missing values. The cJADAS10 disease activity score (23% missing) and the three CHAQ index (disability index (21%), health status (21%), and pain index (21%)) were the variables that had the most missing values. We used a complete dataset when we performed analyses with regard to those specific variable, and the sample size still ranged from 360 to 467.

The parent proxy is a recommended and common type of surrogate to rate the functional disability and disease activity among JIA population, especially for children younger than 8 years old [29]. When comparing with the CHAQ parent-proxy version, we found the EQ-5D-Y-5L parent-proxy version is a valid instrument to measure HRQL of the JIA population. However, it is unclear whether the EQ-5D-Y-5L parent-proxy version performs better or worse compared to the self-reported version. In other pediatric disease areas, findings were diverse in terms of the agreement between the parent-proxy version and self-reported version of a PROM [59, 60]. It is important to examine the parent-patient agreement of the EQ-5D-Y-5L among JIA population in future research.

## Conclusion

This research indicates that the EQ-5D-Y-5L proxy version can be appropriately used among patients with JIA, as it demonstrated a low ceiling effect, good construct validity and informativity. Future research is recommended exploring responsiveness and comparing its psychometric performances with EQ-5D-Y-3L and EQ-TIPs, and other JIA-specific HRQL measures.

## Electronic supplementary material

Below is the link to the electronic supplementary material.

Supplementary Material 1

## Footnotes

1 During one patient visit, this patient may be assigned to multiple cohorts based on his/her condition.

2 10 categories: oligoarticular (number of affected joints < 5) JIA (not classified yet: < 6 months), extended oligoarticular JIA, persistent oligoarticular JIA, polyarticular (number of affected joints > 4) JIA rheumatoid factor (RF) negative, polyarticular JIA RF positive, systemic JIA (arthritis with systemic features), enthesitis-related JIA (arthritis with the enthesitis features), psoriatic JIA (arthritis with the psoriatic feature), undifferentiated JIA (does not fulfill the criteria for any JIA subtype or fulfills criteria for more than one subtype), and other.

3 For example, the active joint count categorical variables had three levels: “no active joints”, “1–5 active joints” and “6 or more active joints”. We hypothesized that patients with 1–5 active joints should have worse HRQL compared to those with no active joints, and patients with 6 or more active joints should have worse HRQL compared to those with 1–5 active joints.
