# Supplementary material for: Validation of the EQ-5D-Y-5L parent-proxy version among children with juvenile idiopathic arthritis
Source: Qual Life Res. 2024 Aug 14;33(10):2677–91. doi: 10.1007/s11136-024-03682-4 (PMC11452410; doi:10.1007/s11136-024-03682-4)
Supplement: Supplementary file 1 — Supplementary Material 1 [file 11136_2024_3682_MOESM1_ESM.docx]

## Appendix 1: Demonstrative figures on the relationship between the EQ-5D-Y-5L and the CHAQ

Similar or the same constructs Might be correlated Different constructs (Not linked)

**Arising:**

- - Stand up from a low chair or floor
- - Get in and out of bed or stand up in crib

**Walking:**

- - Walk outdoors on flat ground
- - Climb up five steps

**Eating:**

- - Cut his/her own meat
- - Lift a cup or glass to mouth
- - Open a new cereal box

**Hygiene:**

- - Wash and dry entire body
- - Take a tub bath (get in & out of tub)
- - Get on and off the toilet or potty chair
- - Brush teeth
- - Comb/brush hair

**EQ-5D-Y-5L:**

Mobility

Looking after myself

Usual activities

Pain or discomfort

Worried, sad, or unhappy

**EQ-5D-Y-5L:**

Mobility

Looking after myself

Usual activities

Pain or discomfort

Worried, sad, or unhappy

**Reach:**

- - Reach and get down a heavy object such as a large game or books from just above his/her head
- - Bend down to pick up clothing or a piece of paper from the floor
- - Pull on a sweater over his/her head
- - Turn neck to look back over shoulder
- - Comb/brush hair

**Grip:**

- - Write or scribble with pen or pencil
- - Open car doors
- - Open jars which have been previously opened
- - Turn faucets on and off
- - Push open a door when he/she has to turn a door knob

**Activities:**

- - Run errands and shop
- - Get in and out of car or toy car or school bus
- - Ride bike or tricycle
- - Do household chores (e.g., wash dishes, take out trash, vacuuming, yardwork, make bed, clean room)
- - Run and play

**Pain index**

**Dressing and grooming:**

- - Dress, including tying shoelaces and doing buttons
- - Shampoo his/her hair
- - Remove socks
  - Cut fingernails

## Appendix 2: The formation of known groups

| Factors | Format of variables (Sources) | Association between factors and HRQL | Known groups | The cutoff was based on… | |
| --- | --- | --- | --- | --- | --- |
| Disease subtype | Oligoarticular vs. Non-Oligoarticular [42]  Oligoarticular vs. Polyarticular  vs. Systemic JIA [35, 43]  Oligoarticular vs. Polyarticular  vs. Enthesitis or Psoriatic JIA [41] | Patients with oligoarticular JIA are associated with better higher HRQL compared to other subtypes | **Oligoarticular** (Oligoarticular JIA (not classified yet: < 6 months), Persistent Oligoarticular JIA, and Extended Oligoarticular JIA)  vs. **Other JIA subtypes** (Polyarticular JIA RF negative, Polyarticular JIA RF positive, Enthesitis-related arthritis, Psoriatic arthritis, Undifferentiated JIA, Systemic JIA) | |  |
| Disease duration | Disease duration < 1 year - binary [42] Disease duration - continuous [44] | Patients with longer disease duration are associated with better HRQL | **More than 12 months**  vs. **Up to 12 months** | Categories presented in the 1st UCAN paper [29] | |
| Disease activity | Active/inactive disease status - binary [42] | Patients with active disease are associated with worse HRQL | **Inactive**  vs. **Active** |  | |
|  | The number of active joints - continuous [45] | Patients with more active joints are associated with worse HRQL | **No active joints**  vs. **1-5 active joints**  vs. **6 or more active joints** | 25% and 75% quantile | |
|  | Morning stiffness more than 15 minutes - binary [47] | Patients with joint stiffness for ≥ 15 minutes are associated with worse HRQL | **No stiffness (stiffness < 15 minutes)**  vs. **Having stiffness** | | |
|  | High disease activity based on cJADAS71 - binary [42]  High disease activity based on cJADAS27 - binary [46] | Patients with high disease activity are associated with worse HRQL | **Inactive disease**  vs. **Minimal disease activity**  vs. **Moderate disease activity**  vs. **High disease activity** | Published cJADAS cutoff points^1^ [31] | |
|  | Physician disease activity (VAS score) - continuous [45] | Patients with high disease activity are associated with worse HRQL | **PGA: 0 (no activity) - 0.9**  vs. **PGA: 1-4**  vs. **PGA: 4.1-10 (maximum activity)** | 25% and 75% quantile | |

| Pain | CHAQ pain index - continuous [45]; CHAQ pain >30 - binary [46] | Patients suffering more pain are associated with worse HRQL | **CHAQ pain index: 0 (no pain)-10**  vs. **11-68**  vs. **69-100 (very severe pain)** | 25% and 75% quantile |
| --- | --- | --- | --- | --- |
|  | Joint pain - binary [42] | Patients suffering joint pain are associated with worse HRQL | **No joint pain**  vs. **Having joint pain** | |
| Functional disability | CHAQ disability index - continuous [45] Severe disability based on CHAQ disability index - binary [42]  Having disability based on the CHAQ disability index - binary [46] | Patients with more functional disabilities are associated with worse HRQL | **No to mild disability (disability index < 0.75)**  vs. **Moderate to severe disability (disability index ≥ 0.75)** | Disability index greater than 0.75 ^2^ [57] |
| Wellbeing | CHAQ VAS score - continuous [45, 46] | Patients with worse well-being are associated with worse HRQL | **CHAQ health status: 0 (best health)-10**  vs. **10-62**  vs. **63-100** | 25% and 75% quantile |

Note: 1. This guideline only provides JADAS cutoff points for poly- and oligo-arthritis to infer disease activity. While we consult clinicians about cutoffs for other JIA subtypes, we tentatively use the cutoffs for polyarthritis on all subtypes other than oligoarthritis. Therefore, the cutoff point for oligoarthritis would be thus: Inactive disease (0 < cJADAS10 score ≤ 1.1), Minimal disease activity (1.1 < cJADAS10 score ≤ 4), Moderate disease activity (4 < cJADAS10 score ≤ 12.0), High disease activity (cJADAS10 score >12.0); and the cutoff point for other subtypes would be thus: Inactive disease (0 < cJADAS10 score ≤ 2.5), Minimal disease activity (2.5 < cJADAS10 score ≤ 5.0), Moderate disease activity (5.0 < cJADAS10 score ≤ 16.0), High disease activity (cJADAS10 score > 16.0).

2. A CHAQ disability index score of 0.75 indicates that this patient has some difficulty (mild disability) in 6 domains, or much difficulty (moderate disability) in 3 domains, or was unable to perform the task (severe disability) in 2 domains. This is a validated cutoff to represent moderate to severe disability.

## Appendix 3: Convergent-divergent validity results: Correlation between EQ-5D-Y-5L and CHAQ dimensions, presented by “theoretically strongly correlated”, “theoretically moderately correlated”, and “theoretically weakly correlated or no correlation” categories (Sensitivity analyses including patients aged 1-3: 433 patient visits representing 388 patients)

|  | Dressing and grooming | Arising | Eating | Walking | Hygiene | Reach | Grip | Activities | Pain index |
| --- | --- | --- | --- | --- | --- | --- | --- | --- | --- |
| **Theoretically strongly correlated** |  |  |  |  |  |  |  |  |  |
| Mobility |  | **0.60**  (0.53-0.66) |  | **0.69**  (0.63-0.73) |  |  |  | **0.64**  (0.58-0.69) |  |
| Looking after myself | **0.61**  (0.55-0.67) |  |  |  | **0.60**  (0.54-0.66) | 0.49  (0.42-0.56) |  |  |  |
| Doing usual activities |  |  |  |  |  | **0.53**  (0.45-0.59) | 0.36  (0.28-0.44) | **0.67**  (0.62-0.72) |  |
| Having pain or discomfort |  |  |  |  |  |  |  |  | **0.73**  (0.68-0.77) |
| Feeling worried, sad, or unhappy |  |  |  |  |  |  |  |  |  |
| **Theoretically moderately correlated** |  |  |  |  |  |  |  |  |  |
| Mobility |  |  |  |  | 0.48  (0.40-0.55) | 0.43  (0.35-0.50) |  |  | **0.57**  (0.50-0.63) |
| Looking after myself |  | 0.48  (0.40-0.55) | 0.35  (0.26-0.43) | 0.45  (0.37-0.52) |  |  | 0.36  (0.27-0.44) |  | 0.36  (0.27-0.44) |
| Doing usual activities | 0.40  (0.32-0.48) | **0.58**  (0.52-0.64) |  | **0.57**  (0.50-0.63) | 0.47  (0.39-0.54) |  |  |  | **0.63**  (0.57-0.68) |
| Having pain or discomfort |  | **0.55**  (0.49-0.62) |  | **0.52**  (0.45-0.58) |  | 0.49  (0.41-0.56) | *0.33*  (0.24-0.41) | **0.58**  (0.51-0.64) |  |
| Feeling worried, sad, or unhappy |  |  |  |  |  |  |  |  |  |
| **Theoretically weakly correlated or no correlation** | |  |  |  |  |  |  |  |  |
| Mobility | 0.37  (0.29-0.45) |  | *0.11*  (0.01-0.20) |  |  |  | *0.17*  (0.07-0.26) |  |  |
| Looking after myself |  |  |  |  |  |  |  | 0.43  (0.35-0.50) |  |
| Doing usual activities |  |  | *0.23*  (0.14-0.32) |  |  |  |  |  |  |
| Having pain or discomfort | 0.37  (0.28-0.45) |  | *0.19*  (0.10-0.28) |  | 0.41  (0.33-0.49) |  |  |  |  |
| Feeling worried, sad, or unhappy | *0.31*  (0.22-0.40) | *0.34*  (0.25-0.41) | *0.24*  (0.14-0.32) | *0.30*  (0.21-0.38) | *0.31*  (0.23-0.40) | 0.37  (0.29-0.45) | *0.34*  (0.25-0.42) | 0.43  (0.35-0.50) | 0.45  (0.37-0.52) |

Note: Bolded and underlined values represent strong associations. Italicized values represent no to weak associations. All correlation coefficients were significant at p<0.001.

Appendix 4: Known group analysis in terms of EQ-5D-Y-5L level summary scores (non-parametric version)

|  | Base-case (excluding aged 1-3) | | | |  | Sensitivity (including aged 1-3) | | | |
| --- | --- | --- | --- | --- | --- | --- | --- | --- | --- |
|  | Patient visits=467; Patient N=407 | | | |  | Patient visits=540; Patient N=472 | | | |
| Known groups  *(Sample sizes n= base case, sensitivity analysis)* | Median (IQR) | Diff. | Effect size | p-value |  | Median (IQR) | Diff. | Effect size | p-value |
| JIA classification (*n=425, 490*) |  |  |  |  |  |  |  |  |  |
| Oligoarticular (*n=187, 231*) | 9 (6-11) |  |  |  |  | 9 (6-12) |  |  |  |
| Other subtypes (*n=238, 259*) | 10 (7-13) | 1 | 0.20 (0.09-0.31) | <0.001 |  | 10 (7-13) | 1 | 0.14 (0.04-0.24) | 0.007 |
| Duration of disease at the time of the visit (*n=420, 484*) |  |  |  |  |  |  |  |  |  |
| More than 12 months (*n=193, 202*) | 8 (6-11) |  |  |  |  | 8 (6-11) |  |  |  |
| Up to 12 months (*n=227, 282*) | 10 (8-13) | 2 | 0.26 (0.15-0.36) | <0.001 |  | 10 (8-13) | 2 | 0.28 (0.18-0.38) | <0.001 |
| Disease activity: Disease status (*n=467, 540*) |  |  |  |  |  |  |  |  |  |
| Inactive (*n=79, 82*) | 6 (5-7) |  |  |  |  | 6 (5-7) |  |  |  |
| Active (*n= 388, 458*) | 10 (8-13) | 4 | 0.71 (0.61-0.80) | <0.001 |  | 10 (8-13) | 4 | 0.72 (0.62-0.80) | <0.001 |
| Disease activity: Active joint count (*n=460, 532)* |  |  |  |  |  |  |  |  |  |
| No active joints (*n=96, 100*) | 6 (5-8) |  |  |  |  | 6 (5-8) |  |  |  |
| 1-5 active joints (*n = 253, 310*) | 10 (7-12) | 4 | 0.52 (0.39-0.63) | <0.001 |  | 10 (7-12) | 4 | 0.54 (0.42-0.64) | <0.001 |
| 6 or more active joints (*n= 111,122*) | 11 (9-14) | 1 | 0.21 (0.08-0.33) | 0.01 |  | 11 (9-14) | 1 | 0.18 (0.06-0.29) | 0.02 |
| Disease activity: Presence of morning joint stiffness (*n=467, 540*) |  |  |  |  |  |  |  |  |  |
| No stiffness (stiffness < 15 min) (*n=328, 379*) | 8 (6-11) |  |  |  |  | 9 (6-12) |  |  |  |
| Having stiffness (*n=139, 161*) | 11 (9-14) | 3 | 0.34 (0.23-0.43) | <0.001 |  | 11 (9-14) | 2 | 0.30 (0.21-0.40) | <0.001 |
| Disease activity: Disease activity based on cJADAS10 scores (*n=360, 420* ) |  |  |  |  |  |  |  |  |  |
| Inactive disease (*n=51, 53*) | 5 (5-7) |  |  |  |  | 5 (5-7) |  |  |  |
| Minimal disease activity (*n=32, 42*) | 7 (6-10) | 2 | 0.43 (0.18-0.62) | 0.18 |  | 7 (6-10) | 2 | 0.45 (0.24-0.63) | 0.10 |
| Moderate disease activity (*n=182, 216*) | 10 (8-12) | 3 | 0.49 (0.29-0.65) | <0.001 |  | 10 (8-12) | 3 | 0.50 (0.34-0.64) | <0.001 |
| High disease activity (*n=95, 109*) | 12 (10-15) | 2 | 0.33 (0.19-0.46) | <0.001 |  | 12 (10-16) | 2 | 0.32 (0.19-0.43) | <0.001 |
| Disease activity: Physician Global Assessment of disease activity (PGA) (*n=467, 540*) |  |  |  |  |  |  |  |  |  |
| PGA: 0 (no activity) - 0.9 (*n=85, 88*) | 6 (5-7) |  |  |  |  | 6 (5-7) |  |  |  |
| PGA: 1-4 (*n= 289, 340*) | 10 (7-12) | 4 | 0.60 (0.48-0.69) | <0.001 |  | 10 (7-12) | 4 | 0.61 (0.50-0.70) | <0.001 |
| PGA: 4.1-10 (maximum activity) (*n= 93, 112*) | 11 (9-15) | 1 | 0.28 (0.15-0.40) | <0.001 |  | 12 (9-15) | 2 | 0.29 (0.17-0.40) | <0.001 |
| Pain: CHAQ pain index (*n=370, 433*) |  |  |  |  |  |  |  |  |  |
| 0 (no pain)-10 (*n=89, 102*) | 6 (5-7) |  |  |  |  | 6 (5-7) |  |  |  |
| 11-68 (*n= 189, 226*) | 10 (8-12) | 4 | 0.76 (0.64-0.83) | <0.001 |  | 10 (8-12) | 4 | 0.69 (0.58-0.78) | <0.001 |
| 69-100 (very severe pain) (*n=92, 105*) | 13 (10-16) | 3 | 0.55 (0.42-0.65) | <0.001 |  | 14 (11-17) | 4 | 0.54 (0.42-0.64) | <0.001 |
| Pain: Presence of joint pain (*n=467, 540*) |  |  |  |  |  |  |  |  |  |
| No joint pain (*n= 321, 376*) | 9 (6-12) |  |  |  |  | 10 (6-12) |  |  |  |
| Having joint pain(*n=146, 164*) | 10 (7-12) | 1 | 0.13 (0.02-0.23) | 0.02 |  | 10 (8-13) | 0 | 0.09 (0.006-0.19) | 0.08 |
| Function disability: CHAQ disability index (*n=370, 433*) |  |  |  |  |  |  |  |  |  |
| No to moderate disability (*n=220,253*) | 7 (6-10) |  |  |  |  | 7 (6-10) |  |  |  |
| Severe disability (disability index ≥ 0.75) (*n=150, 180*) | 12 (10-15) | 5 | 0.75 (0.66-0.81) | <0.001 |  | 12 (10-16) | 5 | 0.71 (0.63-0.78) | <0.001 |
| Wellbeing: CHAQ health status (*n=369, 432*) |  |  |  |  |  |  |  |  |  |
| 0 (best health)-10 (*n=99, 116*) | 6 (5-7) |  |  |  |  | 6 (5-7) |  |  |  |
| 10-62 (*n=180, 215*) | 10 (8-12) | 4 | 0.74 (0.64-0.83) | <0.001 |  | 10 (8-12) | 4 | 0.72 (0.62-0.79) | <0.001 |
| 63-100 (*n=90, 101*) | 13 (10-16) | 3 | 0.49 (0.35-0.60) | <0.001 |  | 14 (10-17) | 4 | 0.45 (0.32-0.57) | <0.001 |

Note: JIA Juvenile Idiopathic Arthritis, cJADAS10 clinical juvenile arthritis disease activity score-10, CHAQ Childhood Health Assessment Questionnaire

1. When the known group has three or more levels, we compared each level with the less severe level previous.

2. Effect size was estimated by cliff’s delta.

3. The p-value represented by Mann-Whitney test (binary variable) or Dunn’s test (post-hoc test for the Kruskal-Wallis, variables with 3+ levels, with Bonferroni correction).
